# Siglec-5 is an inhibitory immune checkpoint molecule for human T cells

**DOI:** 10.1111/imm.13470

**Published:** 2022-04-01

**Authors:** Aleksandra Vuchkovska, David G. Glanville, Gina M. Scurti, Michael I. Nishimura, Paula White, Andrew T. Ulijasz, Makio Iwashima

**Affiliations:** 1 Department of Microbiology and Immunology, Stritch School of Medicine, Loyola University Chicago, Maywood, Illinois, USA; 2 Van Kampen Cardiopulmonary Research Laboratory, Stritch School of Medicine, Loyola University Chicago, Maywood, Illinois, USA; 3 Department of Surgery, Stritch School of Medicine, Loyola University Chicago, Maywood, Illinois, USA; 4 Department of Gynecology and Obstetrics, Stritch School of Medicine, Loyola University Chicago, Maywood, Illinois, USA

**Keywords:** Group B Streptococcus, immune checkpoint, immunotherapy, Siglec, T cell

## Abstract

Sialic acid-binding immunoglobulin-type lectins (Siglecs) are a family of immunoglobulin-type lectins that mediate protein-carbohydrate interactions via sialic acids attached to glycoproteins or glycolipids. Most of the CD33-related Siglecs (CD33rSiglecs), a major subfamily of rapidly evolving Siglecs, contain a cytoplasmic signaling domain consisting of the immunoreceptor tyrosine-based inhibitory motif (ITIM) and immunoreceptor tyrosine-based switch motif (ITSM) and mediate suppressive signals for lymphoid and myeloid cells. While most CD33rSiglecs are expressed by innate immune cells, such as monocytes and neutrophils, to date, the expression of Siglecs in human T cells has not been well appreciated. In this study, we found that Siglec-5, a member of the CD33rSiglecs, is expressed by most activated T cells upon antigen receptor stimulation. Functionally, Siglec-5 suppresses T cell activation. In support of these findings, we found that Siglec-5 overexpression abrogates antigen receptor induced activation of NFAT and AP-1. Furthermore, we show that GBS β-protein, a known bacterial ligand of Siglec-5, reduces the production of cytokines and cytolytic molecules by activated primary T cells in a Siglec-5 dependent manner. Our data also show that some cancer cell lines express a putative Siglec-5 ligand(s), and that the presence of soluble Siglec-5 enhances tumor-cell specific T cell activation, suggesting that some tumor cells inhibit T cell activation via Siglec-5. Together, our data demonstrate that Siglec-5 is a previously unrecognized inhibitory T cell immune checkpoint molecule and suggest that blockade of Siglec-5 could serve as a new strategy to enhance anti-tumor T cell functions.

## INTRODUCTION

Sialic-acid-binding immunoglobulin-like lectins (Siglecs), a family of C-type lectins that bind sialylated glycans, are mainly expressed by innate immune cells and have immunomodulatory functions [1]. Sialic acids are derivatives of the sugar neuraminic acid and are attached on the terminal position of glycoproteins or glycolipids. When sialic acids engage the Siglec family of receptors, they modulate the activation and effector functions of Siglec expressing immune cells [2]. Most Siglecs of the rapidly evolving CD33 related subfamily (CD33rSiglec) have immune suppressive functions, which they mediate through the conserved immunoreceptor tyrosine-based inhibitory motifs (ITIMs) or immunoreceptor tyrosine-based switch motifs (ITSMs) [1]. The arrangement of the ITIM and ITSM of inhibitory Siglecs mirrors the arrangement in the well-known inhibitory checkpoint receptor PD-1 [3]. However, little to no expression of CD33rSiglecs has been reported for T cells except for some pathogenic conditions [4–6].

Here, we demonstrate that human activated T cells express Siglec-5. Previous work by others showed that Siglec-5 suppresses the response of innate immune cells, such as monocytes and neutrophils against pathogens [7,8]. For example, some strains of Group B Streptococcus (GBS) engage Siglec-5 via the surface β-protein, an action that reduces bacterial clearance by suppressing bacterial killing and phagocytosis [8,9]. In adults, GBS is a common commensal bacterium in the gastrointestinal and vaginal tract; however, in newborns, GBS is the leading cause of life-threatening sepsis and meningitis [10]. Our data demonstrate that overexpression of Siglec-5 inhibits the activity of T cell receptor (TCR)-induced transcription factors. Furthermore, engagement of Siglec-5 with the GBS β-protein reduces primary T cell activation and leads to decreased production of cytokines and surface antigens. To our surprise, some cancer cell lines express putative ligands that bind Siglec-5. Importantly, T cell responses against the putative ligand positive cancer cell lines are enhanced in the presence of soluble Siglec-5. Together, our data show that Siglec-5 is a novel and previously unidentified inhibitory T cell immune checkpoint molecule.

## RESULTS

### CD33rSiglec expression by human T cells

**FIGURE 1:**
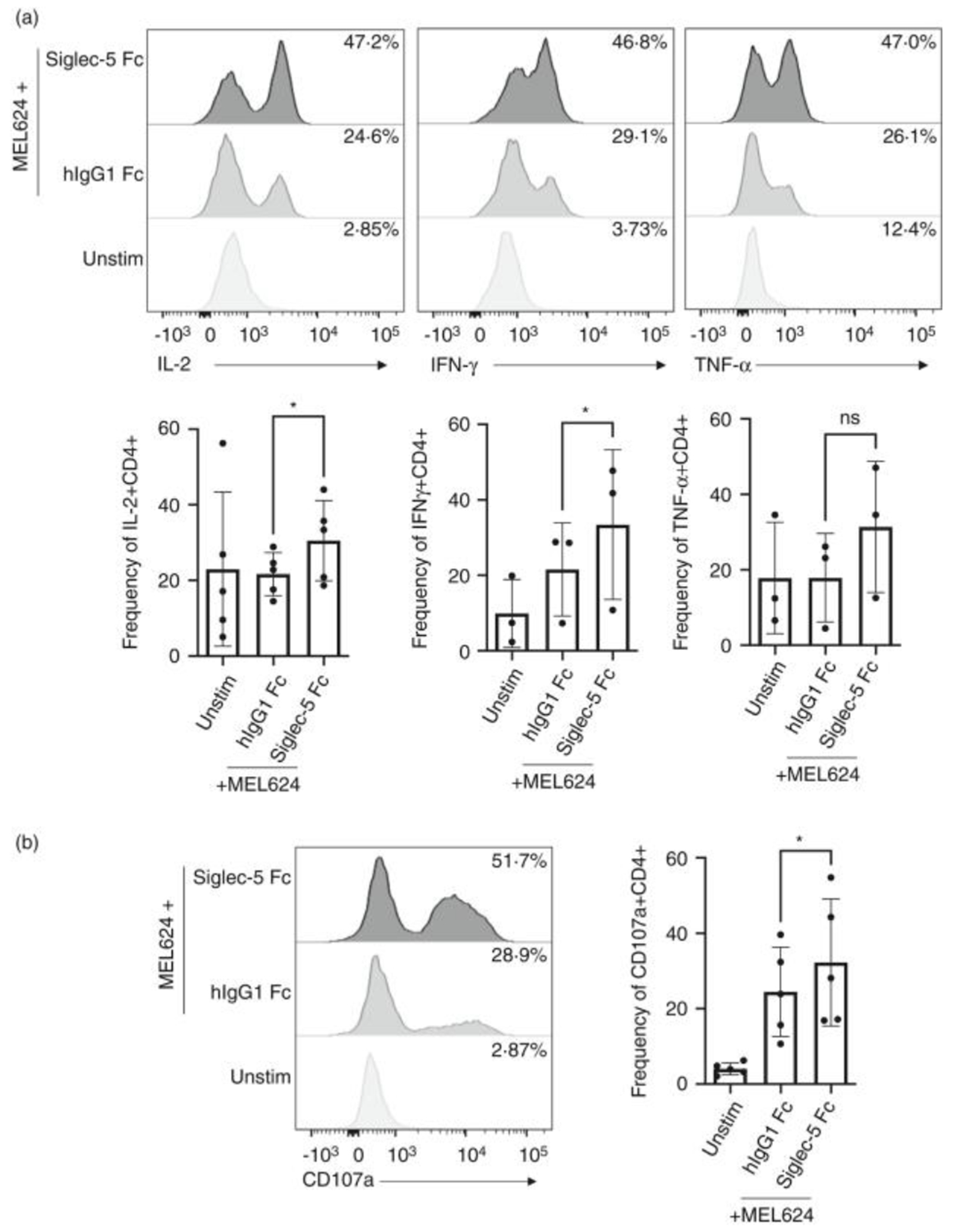
CD33rSiglec expression by resting and activated T cells. Adult peripheral blood or cord blood mononuclear cells were subjected to *in vitro* activation using soluble anti-CD3 and IL-2 for up to 7 days. Cells were split every 2–3 days. (a) Expression of Siglecs-3, –5, –6, –7, –8, –9, –10 was evaluated in the CD4 and CD8 T cell from multiple adult donors. (b) Representative plots and (c) summary for multiple adult donors (each dot represents an individual donor) of Siglec-5 expression within CD4 and CD8 T cells. (d) Summary for multiple cord blood donors of Siglec-5 expression within CD4 and CD8 T cells. (e) Summary for multiple cord blood donors (*n* = 3) for expression of Siglec-5 and PD-1 and (f) Siglec-5 and CTLA-4 within CD4 and CD8 T cells

Innate immune cells express multiple CD33rSiglecs[1]. In contrast, little to no expression is reported in T cells [11], with the exception of Siglec-7 and −9, which are expressed by small populations of CD8+ T cells and mediate direct inhibition of TCR signaling [12]. To obtain a global perspective on Siglec expression by T cells, we assessed the expression of the CD33rSiglec family members (Siglec-3, −5, −7, −8, —9 and −10) in both resting and stimulated cells. As a positive control for the staining, we also included human monocytes from adult PBMCs (Figure S1). Among the Siglecs we tested, Siglec-5 showed a robust activation-associated pattern of expression by both CD4 and CD8 T cells (Figure 1A,B). In contrast, we observed minimal levels of Siglec-6, −8, −9 and −10 expression and a low level of Siglec-3 (both CD4 and CD8) and Siglec-7 (CD8) expression (Figure 1A). Siglec-3 and Siglec-7 expression in the T cells was significantly lower compared to Siglec-5 and did not demonstrate activation-associated pattern of expression. As a result of these observations, we therefore focused on the expression and function of Siglec-5 in human T cells.

Cell surface expression of Siglec-5 was detected at 48hrs post activation in both adult and cord blood CD4 and CD8 T cells (Figure 1C,D). In adult T cells, Siglec-5 expression peaked at 72hrs post-activation, upon which it begun to gradually decrease and was lost by day 7 post-activation (Figure 1B,C). Cord blood T cells also demonstrated Siglec-5 expression after stimulation. The surface expression of Siglec-5 by cord blood T cells reached peak expression earlier than in adult T cells, at 48hrs post stimulation (Figure 1D). Siglec-5 expression kinetics overlapped with the co-inhibitory checkpoint receptor PD-1 (Figure 1E and Figure S2A), while CTLA-4 preceded Siglec-5 and its expression started one day post-stimulation (Figure 1F and Figure S2B). Siglec-5’s expression kinetics closely overlapped with OX-40 expression (Figure S3B), but lagged behind ICOS expression, which peaks at day 1 post-stimulation (Figure S3A). 4-1BB expression is much lower in CD4 T cells compared to Siglec-5, and CD8 T cells express 4-1BB earlier than Siglec-5 (Figure S3C).

### Post-translational regulation of surface expression of Siglec-5

**FIGURE 2:**
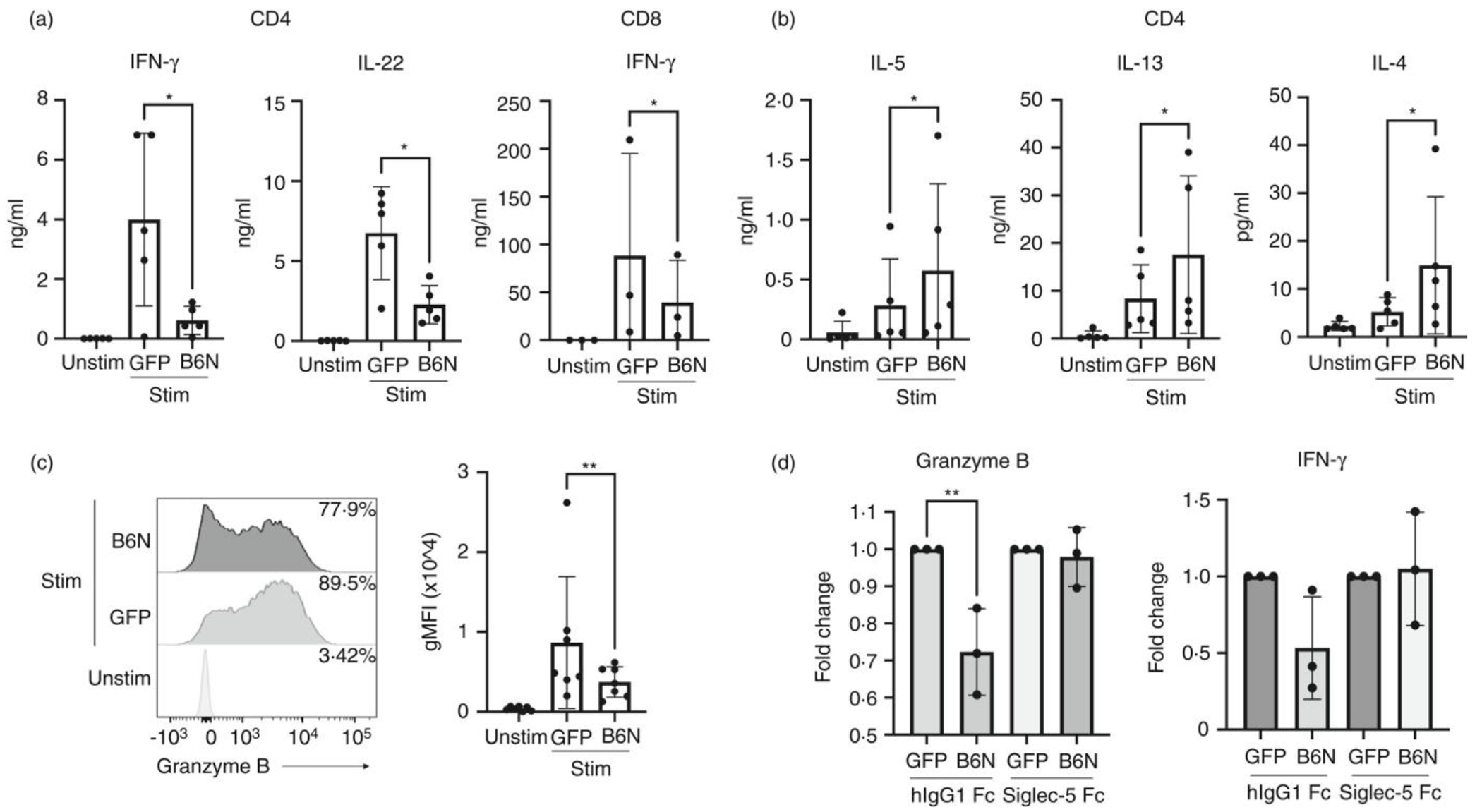
Verification of Siglec-5 expression by human T cells. (a) CD3+ T cells were enriched from adult PBMCs and cultured with plate bound anti-CD3 and anti-CD28 stimulation and IL-2, for up to 4 days. mRNA was prepared at each time point and Siglec-5 mRNA was detected using qPCR. (b) Representative blot (*n* = 3) of CD3+ T cells enriched from adult PBMCs cultured with plate bound anti-CD3 and anti-CD28 stimulation and IL-2, for up to 3 days. At each time point, cell lysates were prepared using denaturing non-reducing conditions. (c) and (d) Cord blood T cells were stimulated with soluble anti-CD3 and IL-2 for 2 days. Cells were lysed with mild detergent (0·5% NP-40) and proteins were immunoprecipitated (IP) using anti-Siglec-5/14 mAb (clone 1A5) or mIgG1 isotype CNBr conjugated beads. As controls, Jurkat T cells were transfected with Siglec-5, Siglec-14 or empty vector and whole cell lysates were prepared. Where indicated, IP-ed proteins were treated with PNGase F to remove N-linked glycosylation. All samples were run on SDS-PAGE and transferred to PVDF membranes. Membranes were blotted with anti-Siglec5/14 polyclonal antibody. Representative membranes of 2 independent repeats

We next performed quantitative RT-PCR to determine the amount of *siglec-5* mRNA in resting and stimulated adult T cells. In contrast to the surface expression, peak mRNA expression of *siglec-5* was observed within 24 h after stimulation and rapidly decreased after that (Figure 2A). The time gap between mRNA and surface Siglec-5 expression suggested that there is a post-transcriptional process that controlled the surface expression of Siglec-5. Similar regulation has been observed for CTLA-4, which surface expression is tightly controlled by membrane endo/exocytosis [13]. To test how Siglec-5 surface expression is regulated, we determined Siglec-5 protein levels via western blot. Surface expression of Siglec-5 was not detectable on the surface until 48hrs post-stimulation (Figure 1B,C); however, Siglec-5 protein was clearly present in activated T cells as early as 24 h post-stimulation (Figure 2B). These data suggest that there is a post-translational mechanism that controls the surface expression of Siglec-5, possibly by membrane trafficking and/or endo/exocytosis of the protein to the cell surface.

There is a known Siglec family member, Siglec-14, that shares 74% protein sequence homology with Siglec-5 within the ectodomain [14]. Most commercially available antibodies cross-react and recognize both Siglec-5 and Siglec-14. Thus, it is important to confirm that the anti-Siglec-5 antibody (clone 1A5) used for flow cytometric analysis of the surface protein (Figure 1) detects Siglec-5, and not Siglec-14, following T cell activation. As shown in Figure 2B, using samples from activated human T cells and prepared under non-reducing conditions, we detect a major protein band at molecular weight (MW) 150 kD. This band corresponds to the predicted homodimer formed by Siglec-5 [7]. To further confirm that the protein we detected is Siglec-5, and not Siglec-14, we immunoprecipitated proteins from activated primary T cells using anti-Siglec-5/-14 antibody (clone 1A5). As a control, we overexpressed Siglec-5 or Siglec-14 cDNA in Jurkat T cells. The predicted size of the Siglec-5 monomer is 68 kDa, while that of Siglec-14 is 42 kD. The bands we observed from the immunoprecipitated samples (using polyclonal anti-Siglec-5/-14 antibody) corresponded to our control Siglec-5 overexpression in Jurkat T cells. However, both the control and the immunoprecipitated bands ranged around 85 kD (Figure 2C), a higher MW than the predicted 68 kDa for Siglec-5. Because Siglec-5 has at least eight described N-based glycosylation sites [15], we hypothesized that the higher-than-expected MW is due to the glycosylation. Indeed, when we treated the samples with PNGase F to remove N-linked glycosylation, we observed that the bands from the control Siglec-5 overexpressing Jurkat T cells and immunoprecipitated proteins from primary T cells corresponded to the predicted 68 kDa band for Siglec-5 (Figure 2D). Together, the data demonstrate that the cell surface protein we detected by flow cytometry is Siglec 5, and its expression is controlled by a yet unidentified post-translational mechanism.

### Function of Siglec-5 in T cells

**FIGURE 3:**
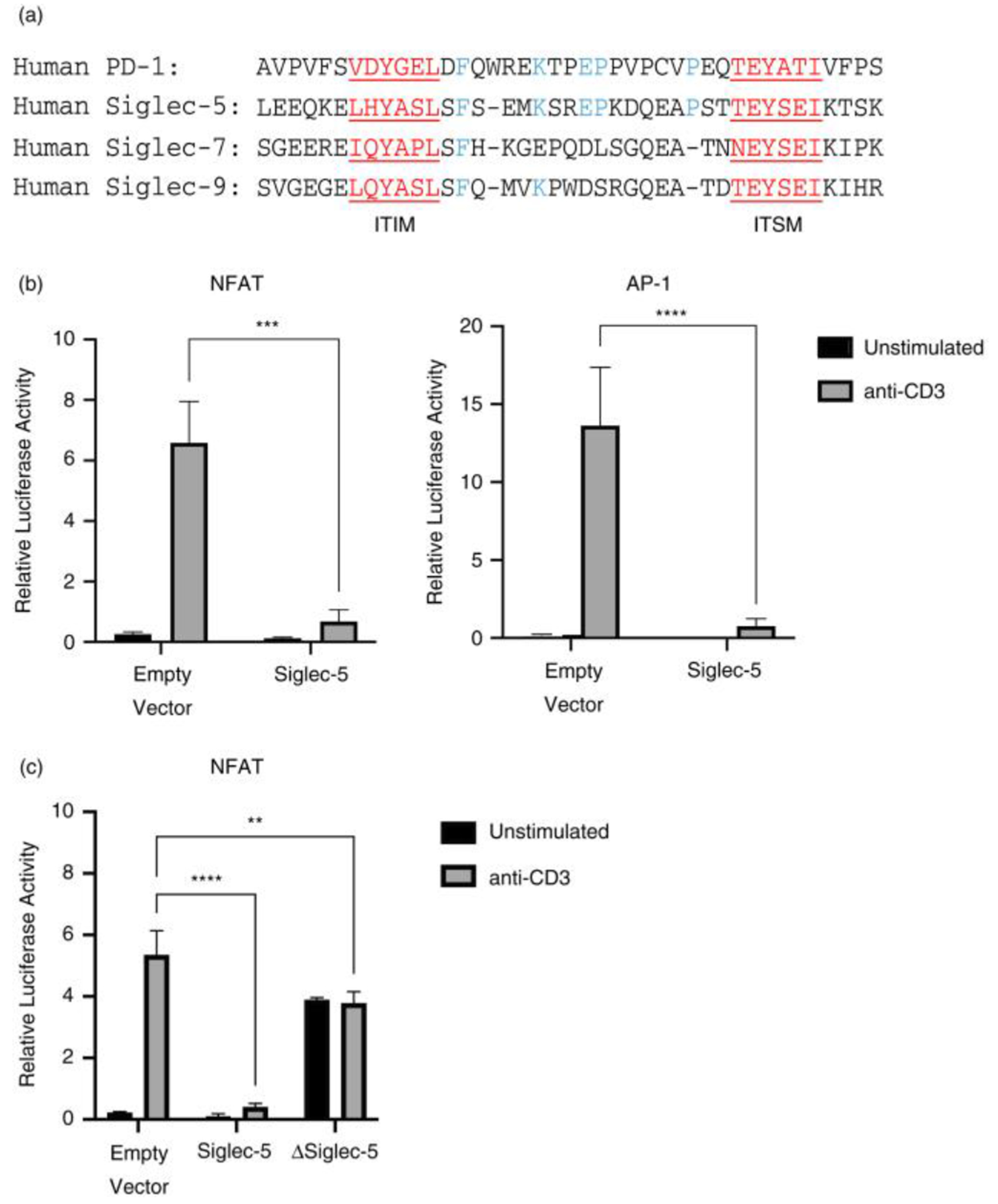
Effect of Siglec-5 overexpression on TCR-induced transcription factor activities. (a) Sequence alignment of the ITIM and ITSM domains of PD-1 and Siglec-5, −7 and −9. Shared protein motifs are highlighted in red, and conserved amino acids are highlighted in blue. (b) and (c) Jurkat T cells were transiently transfected with NFAT or AP-1 luciferase reporter vectors, along with (a) full length Siglec-5 or (b) truncated Siglec-5 (lacking ITIM and ITSM domains). 4hrs post anti-CD3 stimulation, NFAT and AP-1 luciferase activity was measured. Relative luciferase activity was calculated based on Renilla luciferase activity. Representative figures of three independent experiments. Statistical analysis: 2wayANOVA, Tukey’s multiple comparison test, *** *p* < 0·001, **** *p* < 0·0001

In myeloid cells, Siglec-5 mediates its function through the recruitment of Shp1 and Shp2 phosphatases to the ITIM and ITSM present in the cytoplasmic region [16]. Among other inhibitory Siglecs, Siglec-5 has the highest level of similarity with PD-1 within the signaling domain (ITIM and ITSM) (Figure 3A). Based on this information, we hypothesized that Siglec-5 is a negative regulator of T cell activation. To test this, we assessed if overexpression of Siglec-5 reduces TCR-induced activation of the transcription factors NFAT and AP-1. Indeed, overexpression of Siglec-5 significantly reduced TCR-induced NFAT and AP-1 activity following anti-CD3 stimulation (Figure 3B). Siglec-5 overexpression reduced NFAT and AP-1 activity following PMA/Ionomycine stimulation as well (Figure S4A), suggesting that there is a TCR-distal signaling process targeted by Siglec-5. Since ITIM and ITSM are both known to mediate the suppressive functions of PD-1 [17], we hypothesized that Siglec-5 also requires these motifs to suppress NFAT and AP-1. To test this, we generated a truncated Siglec-5 (ΔSiglec-5) that lacks both ITIM and ITSM. If these motifs are required for suppression, we expected to see restoration of NFAT activity in Jurkat T cells expressing ΔSiglec-5. Indeed, we observed a partial restoration in NFAT activity by cells expressing ΔSiglec-5 (Figure 3C and Figure S4B). Unexpectedly, we observed an increase in the basal level of NF-AT activity in unstimulated samples, suggesting that this mutant may be blocking an endogenous molecule that regulates NF-AT in the resting cells. The rescue of NFAT activity in ΔSiglec-5 overexpressing Jurkat T cells was not due to differences in protein levels, as surface Siglec-5 expression between the wild-type and mutant Siglec-5 was comparable (Figure S5).

### Role of Siglec-5 in Group B Streptococcus β-protein mediated suppression of T cell activation

**FIGURE 4:**
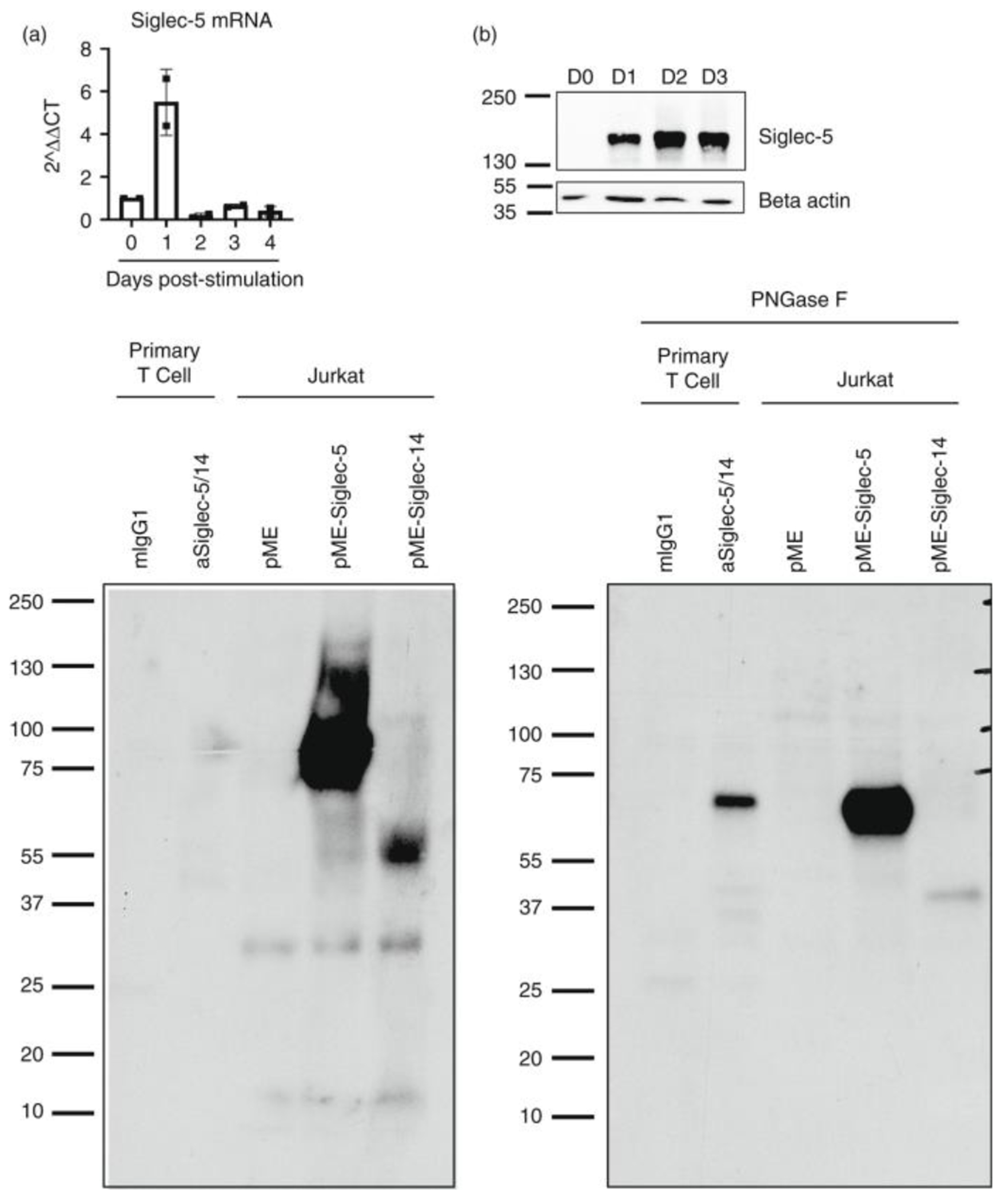
Engagement of Siglec-5 with β-protein and its effect on T cell effector functions. Naïve CD4 or CD8 T cells were cultured with plate bound anti-CD3 and anti-CD28 stimulation in the presence of IL-2 for 3 days. Cells were than harvested and re-stimulated with plate bound anti-CD3, anti-CD28 and B6N::sfGFP or sfGFP. Supernatants and cells from the 3 days cultures were collected and used for (a) and (b) cytokine bead array analysis and (c) measurement of Granzyme B production. Statistical analysis: Ratio paired two-tailed t test; *p < 0·05, **p < 0·01. (d) Day 3 stimulated CD4 T cells were re-stimulated with plate bound anti-CD3, anti-CD28 and B6N::sfGFP or sfGFP pre-incubated with hIgG1 Fc or Siglec-5 Fc at equimolar ratios. At day 3 post-re-stimulation, cytokine production and Granzyme B expression were measured. For each condition, treatments were normalized to unstimulated values first. Fold change was than calculated as the ratio of cells stimulated in the presence of B6N::sfGFP vs sfGFP. Statistical analysis: one-way ANOVA, Tukey’s multiple comparisons test, **p < 0·01. Each dot represents an individual donor

Pathogens have evolved to evade the immune responses by manipulating immunomodulatory mechanisms[18]. Some strains of Group B Streptococcus (GBS), a major pathogen of newborns [10], express a surface protein called β-protein, which binds Siglec-5 and suppresses innate immune cell responses [19]. The Siglec-5 binding region of the GBS β-protein resides within the N-terminal 152aa and is termed the B6N domain [9]. β-protein/Siglec-5 interactions result in inhibition of phagocytosis and suppression of proinflammatory cytokine production, such as IL-8, by neutrophils and monocytes in response to GBS [8,9,19]. To address the function of Siglec-5 during T cell activation, we tested whether engaging Siglec-5 with the B6N region of the β-protein would inhibit primary T cells activation. We generated a B6N fusion protein with superfolder (sf) Green Fluorescent Protein (GFP) (termed B6N::sfGFP) (Figure S6A). The binding capability of Siglec-5 to B6N::sfGFP, but not control sfGFP, was confirmed using an indirect enzyme-linked immunoassay (ELISA) (Figure S6B). To test if Siglec-5 suppressed T cell activation, we stimulated human CD4 or CD8 T cells in the presence of the Siglec-5 ligand B6N::sfGFP. As shown in Figure 1, Siglec-5 expression is elevated at 3 days post-stimulation. Thus, we stimulated naïve T cells for 3 days using plate bound anti-CD3 and anti-CD28 antibodies. Three days after stimulation, we harvested the cells and restimulated them with plate bound anti-CD3 and anti-CD28 in the presence of B6N::sfGFP, or the control sfGFP, for an additional 3 days. After the secondary stimulation, we assessed cytokine production and effector molecule expression by T cells. The stimulation of CD4 T cells in the presence of B6N::sfGFP reduced production of pro-inflammatory cytokines, IFN-γ and IL-22 (we did not observe IL-17A production under these conditions) (Figure 4A), while it increased production of Th2 type cytokines IL-4, IL-5, and IL-13 (Figure 4B). Similarly, B6N::sfGFP reduced production of IFN-γ by CD8 T cells (Figure 4A). Furthermore, B6N::sfGFP also reduced the expression of Granzyme B by activated CD4 T cells (Figure 4C).

To test whether B6N::sfGFP changed T cell cytokine production in a Siglec-5 dependent manner, we pre-incubated B6N::sfGFP with soluble Siglec-5 Fc chimeric protein before coating it onto plates to reduce the availability of B6N::sfGFP that can bind the endogenous Siglec-5 molecule on T cells. Indeed, when pre-treated with the Siglec-5 Fc protein, B6N::sfGFP did not suppress the activation of T cells, as measured by the expression Granzyme B and IFN-γ (Figure 4D), demonstrating that B6N::sfGFP inhibits T cell activation by interacting with Siglec-5.

### Role of Siglec-5 engagement during the T cell specific anti-tumour response

**FIGURE 5:**
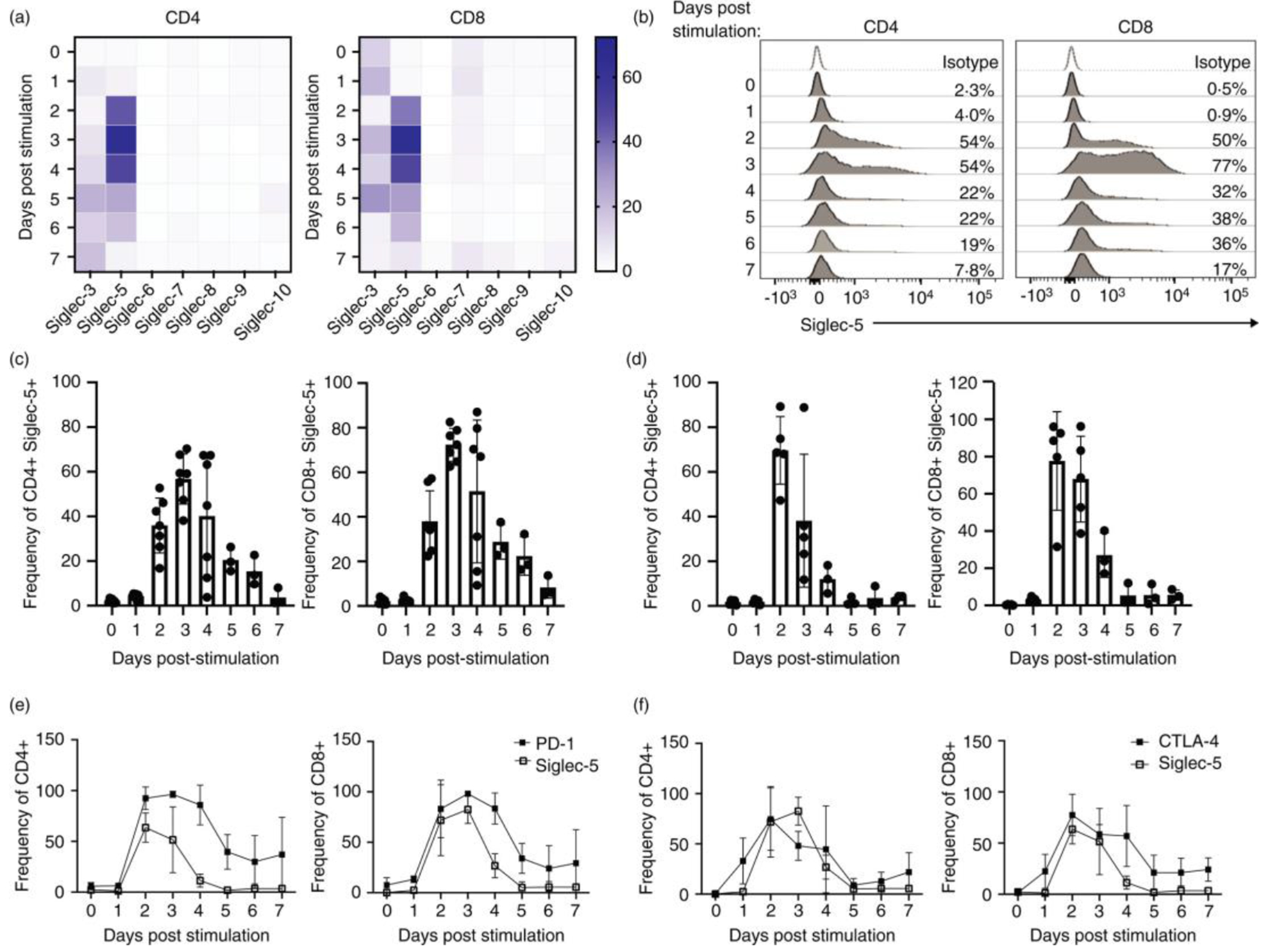
Effect of soluble Siglec-5 Fc on tumor antigen-induced T cell response. 1383i T cells were stimulated with T2 pulsed with tyrosinase peptide. 2 days post-stimulation cells were used for stimulation with MEL624 cancer cell line in the presence of soluble Siglec-5 Fc (10 μg/ml) or control hIgG1 Fc (10 μg/ml) protein. The assay was carried in the presence of Brefeldin A and Monensin. (a) Representative plots and summary of frequency of CD4 T cells expressing IL-2, IFN-γ, and TNF-α at 12hrs post-stimulation. (b) Representative plots and summary of frequency of CD4 T cells expressing CD107a. Statistical analysis: Ratio paired two-tailed *t*-test; *p < 0·05. Each dot represents an individual donor

Siglec-5 expression by tumour infiltrating lymphocytes has been reported [6]. Considering the immunosuppressive functions that inhibitory checkpoint receptors play in anti-tumour immunity, we asked whether Siglec-5 is also involved in the process by which cancers suppress T cell responses. We first investigated if Siglec-5 could interact with human cancer-derived cell lines. Indeed, soluble Siglec-5 bound multiple cancer cell lines, including MEL624, Raji and SUN-475, among others, suggesting that these cell lines express putative ligands for Siglec-5 (Figure S7). To test if the putative ligands expressed by cancer lines could suppress the T cell response, we used engineered human T cells transduced with a TCR (1383i) specific for the melanoma antigen tyrosinase [20]. When 1383i TCR^+^ T cells were stimulated with antigen presenting cells (T2 cells) pulsed with tyrosinase peptide, we observed a robust expression of Siglec-5 (Figure S8). We hypothesized that a soluble form of Siglec-5 (Siglec-5 Fc) would bind the putative Siglec-5 ligands expressed by MEL624 and interfere with the engagement of the endogenous Siglec-5 expressed by activated melanoma antigen specific T cells. To test our hypothesis, we stimulated 1383i TCR + T cells with irradiated T2 cells pulsed with tyrosinase. Two days post primary stimulation, we re-stimulated the 1383i TCR^+^ T cells with the tyrosinase positive cancer line MEL624 [20] in the presence or absence of Siglec-5 Fc protein. Stimulation with MEL624 pre-treated with Siglec-5 Fc led to an increased frequency of IL-2, IFN-γ and TNF-α, producing 1383i TCR^+^ T cells (Figure 5A). Moreover, CD107a, the surface antigen associated with the release of cytotoxic granules, was also significantly increased by 1383i TCR^+^ T cells stimulated by MEL624 pre-treated with Siglec-5 Fc (Figure 5B). In this assay, CD8 T cells re-stimulated with MEL624 did not produce any amounts of cytokines. We hypothesize that in the context of this assay repeated stimulation of CD8 T cells drives them into anergic state. Together, these data showed that engagement of Siglec-5 by putative ligands expressed on the target cells suppressed CD4 T cell activation and further supported our hypothesis that Siglec-5 functions as a novel inhibitory checkpoint molecule for activated human T cells.

## DISCUSSION

In this study, we report that Siglec-5 is an inhibitory surface antigen for human T cell activation. Siglec-5 overexpression inhibits NFAT and AP-1 transcription factor activity. GBS virulence factor β-protein, a known Siglec-5 ligand, inhibits primary T cell cytokine production in a Siglec-5-dependent manner. Furthermore, a soluble form of Siglec-5 enhances cytokine production and cytotoxic granule release by cancer antigen-specific T cells. When taken together, our data demonstrate that Siglec-5 is a previously unrecognized inhibitory checkpoint molecule expressed by activated human T cells. A previous report presents suggestive data of Siglec-5 expression following T cell activation [6]. However, their flow cytometry analysis used the antibody clone 1A5 which cross-reacts with Siglec-5 and Siglec-14. Using the same anti-Siglec-5/Siglec14 antibody clone, 1A5, another group reported Siglec-14 expression by monocytes [21]. Besides their extracellular sequence homology, Siglec-5 and Siglec-14 have opposing roles during immune cell activation, with Siglec-5 mediating inhibitory, and Siglec-14 mediating activating signals. As a result, the surface analysis of Siglec-5 using a cross reactive antibody is not sufficient to draw definitive conclusions. In this report, we used western blotting of immunoprecipitated proteins to confirm that activated T cells express Siglec-5. In comparison to control cells overexpressing Siglec-5 or Siglec-14, we found that immunoprecipitated proteins from activated T cells correspond to Siglec-5, and not Siglec-14 (Figure 2).

Our data show that surface expression of Siglec-5 by activated human T cells coincides with many other co-inhibitory and co-stimulatory molecules, including PD-1 and OX40 (Figure 1E and Figure S3B). The activation of co-stimulatory and co-inhibitory checkpoint receptors depends on their ligand availability [22]. Multiple immune checkpoints are co-expressed by activated T cells, but their ligands are expressed by different cell types suggesting that T cell responses can be differentially regulated based on the spatiotemporal availability of immune checkpoint ligands expressed by antigen presenting cells. In lieu of this knowledge, we are currently investigating the identity and tissue distribution of molecules that bind Siglec-5 to determine potential ligand expression by normal and transformed cells.

It should be noted that the time course of Siglec-5 surface expression does not correspond to the level of total protein expression. In contrast to its surface expression at 48hrs post-stimulation (Figure 1C,D), we can detect *siglec-5* mRNA and total protein expression as early as 24 h post-stimulation (Figure 2A,B). These data suggest that surface translocation and/or exo-/endocytosis of Siglec-5 is tightly controlled, similar to what has been observed for CTLA4 [13]. Further studies are needed to elucidate how trafficking of Siglec-5 to the cell surface is regulated.

The data presented here suggest that Siglec-5 mediates its inhibitory function via the conserved ITIM and ITSM, in a manner that parallels PD-1 [23,24]. Previous studies on the Siglec-5 function in myeloid cells have shown that upon ligand engagement, ITIM and ITSM become phosphorylated, and that these phosphorylated motifs serve as recruitment sites for phosphatases such as Shp1 and Shp2 [8,9] that de-phosphorylate proteins involved in the membrane proximal signaling events. When Siglec-5 was overexpressed in Jurkat T cells, it significantly inhibited the activity of NFAT and AP-1 following TCR stimulation (Figure 3B). However, obtaining such a result in the absence of an added ligand to engage Siglec-5 is enigmatic and raised the question how Siglec-5 initiates its inhibitory effect. Intriguingly, our data also demonstrated that approximately 15% of Jurkat T cells express a putative ligand for Siglec-5 (Figure S8B), suggesting that Siglec-5 was engaged either by the ligands expressed autologously or by neighboring Jurkat T cells. Unexpectedly, Siglec-5 blocked PMA/Ionomycine stimulated NFAT and AP-1 activity as well. These data suggest that Siglec-5 inhibits a broader network of signaling events involved in T cell activation.

Our studies here have also shown that GBS β-protein, a known ligand for Siglec-5, suppressed primary T cell activation in a Siglec-5 dependent manner. Our data show that the N-terminal region of the β- protein, B6N (aa1-152), suppresses the production of pro-inflammatory cytokines such as IFN-γ and IL-22 by primary human CD4 T cells (Figure 4A) while mildly increasing Th2 cytokines IL-4, IL-5 and IL-13, potentially due to the reduced IFN-γ production [25] (Figure 4B). Similarly, B6N region also reduced IFN-γ production by CD8 T cells (Figure 3A). Because IFN-γ plays a crucial role in the clearance of GBS in neonates [26,27], our data here suggest that Siglec-5 mediated T cell suppression in neonates could be a mechanism by which β-protein expressing GBS suppresses the immune response.

Our data also showed that Siglec-5 interacts with a putative endogenous ligand(s) that is expressed by multiple cancer cell lines. Among the cell lines tested, the melanoma cell line MEL624 showed strongest binding to soluble Siglec-5 (Figure S8). Our data show that soluble Siglec-5 increased the frequency of IL-2, IFN-γ and TNF-α by MEL624 tumor antigen specific CD4 T cells. Soluble Siglec-5 also increased the frequency of cells expressing CD107a (also known as LAMP-1), a lysosome-associated molecule that marks cells that have recently released cytotoxic granules (CGs). CGs are specialized lysosomes comprised of granzymes and perforins, which mediate targeted cell lysis when released extracellularly. Taken together, our data suggest that soluble Siglec-5 can increase the ability of T cells to respond to antigen expressing cancer cells.

A limitation of this study is the lack of *in vivo* data. The mouse ortholog of Siglec-5 is Siglec-F [28]. However, besides sharing high sequence homology with Siglec-5, Siglec-F is closer to another CD33rSiglec, Siglec-8, with which it shares similar expression patterns, ligand preferences and functional outcomes [29]. Thus, to study the T cell specific role of Siglec-5 in physiological or pathological conditions, a humanized mouse model needs to be established in the future.

## MATERIALS AND METHODS

### Cell culture

Adult peripheral blood mononuclear cells (PBMCs) were purchased from Key Biologics (Memphis, TN) and came from de-identified adult healthy donors. Cord blood mononuclear cells were isolated from whole umbilical cord blood kindly donated from Loyola University Medical Center. T cells were isolated from mononuclear cells from healthy adult or cord blood via negative selection using the MojoSort CD3+, naïve CD4+ or naive CD8+ T cell enrichment kit (Biolegend, San Diego, CA).

### Flow cytometry

Antibodies used for the flow cytometry analysis were anti-CD4, -CD8, -Siglec 5 (1A5), -Siglec-3, -Siglec-7, -Siglec-9, -Siglec-10, -CD137, -PD-1, -Granzyme B, -OX40, -ICOS, -CTLA-4, -IFN-γ, -TNF-α, -IL-2 and -CD107a, (Biolegend, San Diego, CA). Intracellular markers were analyzed after fixing and perming with 4% paraformaldehyde and permeabilization buffer (50mM NaCl, 0·02% NaN3, 5mM EDTA, 0·5% TritonX, pH7·5), respectively. Data were collected using BD FACSCanto or BD LSRFortessa and analyzed using FlowJo v.10 software.

### Western blot and immunoprecipitation analysis

For western blot analysis, cells were lysed in non-reducing SDS sample buffer (2% SDS, 0·05% bromophenol blue, 62.5 mM Tris-HCl, pH 6·8, 10% glycerol, 10 mM iodoacetamide (IAM)) and boiled 2 × 5min.

For immunoprecipitation of Siglec-5, cord blood mononuclear cells were stimulated using soluble anti-CD3 (200ng/ml) and IL-2 (10 ng/ml) for 2–3 days. 5–6 × 10^7^ cells were lysed in 1ml of lysing buffer containing 0·5% NP-40, 0·15 M NaCl, 5mM EDTA, and protease and phosphatase inhibitors and applied to immunoprecipitation with anti-Siglec5/14 antibody (clone 1A5, Biolegend, San Diego, CA). Immunoprecipitants were analyzed directly or further subjected to de-glycosylation with PNGase-F (NEB, Ipswich, MA) following the manufacturer’s instructions. Membranes were probed with antibodies against anti-Siglec5/14 (polyclonal antibody, R&D, Minneapolis, MN).

### Reporter and expression constructs, luciferase assay

cDNA for Siglec-5 (SinoBiological) and Siglec-14 (IDT, inc.) were subcloned into a pME18 vector. Truncated version of Siglec-5 (pME-tSiglec-5) was generated by the deletion of the cytoplasmic region of Siglec5. Reporter constructs for NFAT-luciferase and AP-1 luciferase, and the assay method were previously described [30].

### Cloning, expression and purification of B6N::sfGFP and sfGFP

The DNA sequence encoding the B6N region (amino acids 1-152) of the Group B Streptococcus β-protein [9] was codon-optimized for expression in *E. coli* and fused to a super-folder green fluorescent protein variant, sfGFP [31], with a flexible linker (G-S-G-G-G-G-S-G-G-G-G-S). The resulting B6N-linker-sfGFP sequence, or the control linker-sfGFP sequence, was ligated into pET15DG1 [32]. 6xHis-tagged proteins were expressed in T7 Express *lysY/I^q^* *E. coli* cells (New England Biolabs, Ipswich, MA), and purified using Ni-NTA resin (Qiagen, Valencia, CA). Protein-containing fractions were further purified by Size Exclusion Chromatography (SEC) using an AKTA Pure FPLC system (General Electric, Boston, MA).

### T cell stimulation with B6N::sfGFP

Naïve CD4^+^ or CD8^+^ T cells were cultured with plate bound anti-CD3 (5 μg/ml) and anti-CD28 (5 μg/ml) stimulation in the presence of IL-2 (10 ng/ml) for 3 days. Cells were than harvested, washed and re-stimulated with plate-bound anti-CD3 (5 μg/ml), anti-CD28 (5 μg/ml) and B6N::sfGFP or sfGFP (166 nM) for additional 3 days.

To test the role of Siglec5 in T cell inhibition by B6N::sfGFP, equimolar amount of B6N::sfGFP or sfGFP were pre-incubated with recombinant hIgG1 Fc or Siglec-5-Fc proteins. The proteins were than coated on 96 well non-tissue cultured plates along with anti-CD3 and anti-CD28, before adding the 3 days stimulated CD4 T cells. Supernatants from 3-day cultures were collected and used for cytokine bead array (CBA) using the LEGENDplex Human Th Cytokine (Biolegend, San Diego, CA).

### Evaluation of putative Siglec-5 ligands expressed by cell lines

Cell lines MEL624, Jurkat, Raji, THP1, U937, T2, SNU-475, HEK293t, A549 were blocked with human Fc receptor blocking solution (Human TruStain FcX, Biolegend) and then incubated with Siglec-5 Fc chimeric protein (Biolegend, San Diego, CA), followed by anti-human IgG Fc antibody staining.

### Human 1383i TCR+T cell stimulation

Human 1383i TCR+T cells are generated as described [33]. To stimulate the 1383i TCR+T cells, T2 cells (1 × 10^6^ cells/ml) (ATCC, Manassas, VA) were pulsed with tyrosinase peptide 368–376 (YMDGTMSQV) (10 μg/ml), irradiated with 4425cGy, and used for culturing with 1383i TCR + T cells at a ratio of 3:1 (1383i T cells: T2). After 2 days of stimulation, 1383i T cells were re-stimulated with MEL624 pre-treated with hIgG1-Fc (10 μg/ml) (Biolegend, San Diego, CA) or Siglec-5-Fc chimeric protein (10 μg/ml) at 1:1 ratio. Cell cultures were carried in the presence of 1x Monensin and 1x BrefeldinA (Biolegend, San Diego, CA). At 12hrs post-stimulation, the expression of CD107a, IFN-γ, TNF-α and IL-2 was evaluated.

## Supplementary Material

Fig. S2

Fig. S1

Fig. S3

Fig. S5

Fig. S4

Fig. S6

Fig. S7

Fig. S8

## Data Availability

All data are available in the main text or the supplementary materials.
